# Supplementary material for: Resolving the full spectrum of human genome variation using Linked-Reads
Source: Genome Res. 2019 Apr;29(4):635–45. doi: 10.1101/gr.234443.118 (PMC6442396; doi:10.1101/gr.234443.118)
Supplement: Supplemental Material [file supp_gr.234443.118_Supplemental_longranger-2.2.zip › longranger-2.2.tar.bz2!/lr2.2-public/lib/stan/cmdstan/stan/doc/api/html/math_2matrix_2exp_8hpp_source.html]

Stan: src/stan/math/matrix/exp.hpp Source File


|  |  |
| --- | --- |
|  | Stan  2.5.0  probability, sampling & optimization |


- Main Page
- Related Pages
- Namespaces
- Classes
- Files

- File List
- File Members

All Classes Namespaces Files Functions Variables Typedefs Enumerations Enumerator Friends Macros Pages

- src
- stan
- math
- matrix

exp.hpp

Go to the documentation of this file.

1 #ifndef STAN\_\_MATH\_\_MATRIX\_\_EXP\_HPP

2 #define STAN\_\_MATH\_\_MATRIX\_\_EXP\_HPP

3

4 #include <stan/math/matrix/Eigen.hpp>

5 #include <boost/math/special\_functions/fpclassify.hpp>

6 #include <limits>

7

8 namespace stan {

9  namespace math {

10

17  template<typename T, int Rows, int Cols>

18  inline Eigen::Matrix<T,Rows,Cols> exp(const Eigen::Matrix<T,Rows,Cols>& m) {

19  return m.array().exp().matrix();

20  }

21

22  //FIXME:

23  //specialization not needed once Eigen fixes issue:

24  //http://eigen.tuxfamily.org/bz/show\_bug.cgi?id=859

25  template<int Rows, int Cols>

26  inline Eigen::Matrix<double,Rows,Cols> exp(const Eigen::Matrix<double,Rows,Cols>& m) {

27  Eigen::Matrix<double,Rows,Cols> mat = m.array().exp().matrix();

28  for (int i = 0, size\_ = mat.size(); i < size\_; i++)

29  if (boost::math::isnan(m(i)))

30  mat(i) = std::numeric\_limits<double>::quiet\_NaN();

31  return mat;

32  }

33

34  }

35 }

36 #endif

boost::math::isnan

bool isnan(const stan::agrad::var &v)

Checks if the given number is NaN.

**Definition:** boost\_fpclassify.hpp:69

stan::math::exp

Eigen::Matrix< T, Rows, Cols > exp(const Eigen::Matrix< T, Rows, Cols > &m)

Return the element-wise exponentiation of the matrix or vector.

**Definition:** exp.hpp:18

Eigen.hpp

size\_

size\_t size\_

**Definition:** dot\_self.hpp:18


---

[ Stan Home Page ]

*© 2011–2014,
Stan Development Team.*
